# Incidence and clinical characteristics of fall-related injuries among older inpatients at a tertiary grade a hospital in Shandong province from 2018 to 2020

**DOI:** 10.1186/s12877-022-03321-y

**Published:** 2022-08-01

**Authors:** Hong Lyu, Yan Dong, Wenhong Zhou, Chuanxia Wang, Hong Jiang, Ping Wang, Yanhong Sun

**Affiliations:** 1 Department of Geriatric Cardiology, Shandong Provincial Hospital Affiliated to Shandong First Medical University, Jinan, 250021 China; 2 Outpatient Department of Geriatrics, Shandong Provincial Hospital Affiliated to Shandong First Medical University, Jinan, 250021 China; 3 Nursing Department, Shandong Provincial Hospital Affiliated to Shandong First Medical University, Jinan, 250021 China; 4 Department of Geriatrics, Shandong Provincial Hospital Affiliated to Shandong First Medical University, Jinan, 250021 China; 5 Department of Respiratory and Critical Care, Shandong Provincial Hospital Affiliated to Shandong First Medical University, Jinan, 250021 China

**Keywords:** Fall-related injuries, Recurrent fall, Incidence, Risk factors, Clinical characteristics, Older inpatients

## Abstract

**Background:**

Falls are an important cause of injury and death of older people. Hence, analyzing the multifactorial risk of falls from past cases to develop multifactorial intervention programs is clinically significant. However, due to the small sample size, there are few studies on fall risk analysis of clinical characteristics of fallers, especially among older hospitalized patients.

**Methods:**

We collected data on 153 inpatients who fell (age ≥ 60 years) from the hospital nursing adverse event reporting system during hospitalization at Shandong Provincial Hospital Affiliated to Shandong First Medical University, China, from January 2018 to December 2020. Patient characteristics at the time of the fall, surrounding environment, primary nurse, and adverse fall events were assessed. The enumeration data were expressed as frequency and percentage, and the chi-squared was performed between recurrent fallers and single fallers, and non-injurious and injurious fall groups.

**Results:**

Cross-sectional data showed 18.3% of the 153 participants experienced an injurious fall. Compared with single fallers, a large proportion of older recurrent fallers more often experienced preexisting conditions such as cerebrovascular disease or taking hypoglycemic drugs. They were exposed to higher risks and could experience at least 3 fall times in 3 months. Besides, the credentials of their responsible nurses were often higher. Factors that increased the risk of a fall-related injury were hypoglycemic drugs (OR 2.751; 95% CI 1.114–6.795), and nursing adverse events (OR 47.571; 95% CI 14.392–157.247). Older inpatients with bed rails (OR 0.437; 95% CI 0.190–1.005) or falling at the edge of the bed (OR 0.365; 95% CI 0.138–0.964) were less likely to be injured than those without bed rails or not falling at the edge of the bed. Fall risks were significantly correlated with more severe fall-related injuries. Older patients with moderate (OR 5.517; CI 0.687–44.306) or high risk (OR 2.196; CI 0.251–19.219) were more likely to experience fall-related injuries than those with low risk.

**Conclusions:**

Older inpatient falls are an ongoing challenge in hospitals in China. Our study found that the incidence of fall-related injuries among inpatients aged ≥ 60 years remained at a minor level. However, complex patient characteristics and circumstances can contribute to fall-related injuries.

This study provides new evidence on fall-related injuries of older inpatients in China. Based on the factors found in this study, regular fall-related injury epidemiological surveys that investigate the reasons associated with the injuries were crucial when considering intervention measures that could refine fall-related injuries. More prospective studies should be conducted with improved and updated multidisciplinary fall risk assessment and comprehensive geriatric assessment as part of a fall-related injury prevention protocol.

## Background

Population aging has become a serious problem in China over the past few years. China’s seventh census in 2020 [1] revealed that people aged ≥ 60 years accounted for 18.7% of the entire population, an increase of 5.44% from the sixth census in 2010. Falls have been considered complex multifactorial phenomena [2], syndromes, new signs of emergence or deterioration of health status [3], and important causes of injury and death of older people [4]. Older individuals injured by falls may experience fractures, disability, reduced functionality and quality of life, increased financial burden, and mortality [5]. Different retrospective studies [6–9] in mainland China reported that the incidence of fall-related injuries varied from 42.42% to 89.74% among inpatients aged ≥ 60 years. At present, most hospitals use fall assessment tools to predict the occurrence of falls; however, these tools cannot address the various causes and risk factors of falls in hospitals. In 2013, according to the National Institute for Health and Care Excellence, fall risk prediction tools should not be used to predict the risk of patients falling in hospitals [10]. Various risk factors for falls have been identified, and many interventions have proven effective, for example, performing different types of exercise, eliminating environmental hazards, and improving visual acuity [11]. Furthermore, analyzing the reasons associated with injuries can be of great clinical significance for developing and implementing tailored strategies to prevent future recurrent injurious falls, which has been considered an urgent public health challenge. However, due to the small sample size, there are insufficient studies on the fall risk analysis of clinical characteristics based on fallers, especially among older hospitalized patients. Therefore, we conducted a cross-sectional study of falls among inpatients aged ≥ 60 years in a comprehensive tertiary grade A hospital in Shandong Province to further explore the associated characteristics that contributed to fall-related injuries. The findings of the present study might contribute to the design of multifaceted strategies to prevent inpatient fall-related injuries.

## Methods

### Participants

We collected data on 153 inpatients (age ≥ 60 years) who fell from hospital nursing adverse event reporting system (AERS) during hospitalization in Shandong Provincial Hospital Affiliated to Shandong First Medical University, China, from January 2018 to December 2020.

Sample size calculation was performed based on the following assumptions: level of confidence, 95%; type I error rate, 5%; and type II error rate, 0.2, with p as the proportion of older inpatient fallers who experienced injurious falls. Since previous studies conducted on injurious falls in hospitals showed that the proportion of fall-related injuries varied from 42.42% to 89.74%, *p*0 = 66% was set by the median. *p* = 50% was considered to have maximum sample size. Based on these assumptions, the actual sample size for the study was computed using the following formula for a single population proportion:

$$n=p\left(1-p\right){\left(\frac{{z}_{1-\frac{\alpha }{2}}+{z}_{1-\beta }}{p-{p}_{0}}\right)}^{2}$$

where n is the sample size, Z1-α/2 is 1.96, Z1-β is 0.842, p is the proportion of older inpatient fallers who experienced injurious falls (0.5), p0 is the proportion of fall-related injuries in previous studies (0.66). Then *n* = 77. A minimum sample size of 77 patients was required in order to precisely analyze the study and increase the power of to draw convincible conclusions.

All the participants met the inclusion and exclusion criteria (Table 1). This study was approved by the Shandong Provincial Hospital Affiliated to Shandong First Medical University Ethics Committee. This is a 3889-bed tertiary grade A hospital that represents the highest level of medical services in China. The fall rate in this hospital was 0.0691–0.0783 falls per 1,000 bed-days from 2018 to 2020 (Table 2). All methods were conducted according to relevant guidelines and regulations.

**Table 1:** Inclusion and exclusion criteria

Inclusion criteria	Exclusion criteria
1. Male or female aged 60 years or older 2. Reported falls from AERS 3. Falls during the hospitalization period 4. Hospitalized in a medical, surgical, gynecological, emergency, or geriatrics ward	1. Falls occurred outside the hospital and the patient went to a medical institution for treatment of the fall-related injury 2. Falls during outpatient visits

**Table2:** Falls rate in hospitalized patients

Statistical period	Denominator	Numerator	Falls rate(per 1,000 bed-days)
Each year	Number of bed-days in the statistical period	Number of falls occurence among hospitalized patients in the same period	
2018	625,550	98	0.0783
2019	657,160	102	0.0776
2020	550,105	76	0.0691

### Data collection

Based on an extensive literature review [12–14], we developed a detailed fall adverse event data collection system to identify the possible factors leading to falls and fall-related injuries. These included the following:

1. Basic patient information, such as sex, age, diagnosis, visual impairment, consciousness, drainage condition, urine frequency or incontinence, diarrhea or fecal incontinence, usage of high-risk drugs, application of catheters, approximate fall occurrence time in a day, and number of falls in 3 months. The fall risk level was obtained closest to the time of the fall. We used the Morse Fall Scale [15] to assess the likelihood of falling and the Barthel Index [16] to assess the patients’ activity of daily living (ADL).
2. Surrounding environment: location, status, with bed rails, with auxiliary tools.
3. Primary nurses: nurse grading, diploma, working years, and position/title.
4. Fall adverse events: nursing adverse event level, fall-related injury classification, fall process description, cause analysis, and rectification measures.

Fall cases were identified using the nursing AERS. A trained researcher, with experience in fall research, collected fall data from (a) the electronic nursing document system, (b) nursing adverse event database, (c) patient’s paper medical record via a standard template, and (d) interviews between nurses and patients or family members. This training and subsequent data collection procedure was supervised by a senior nurse researcher.

### Definition

Falls were defined as “an unexpected event in which the participant comes to rest on the ground, floor, or other lower level.” [17] Fall-related injuries refer to the different degrees of injury caused by falls. Incidence of fall-related injuries refers to the proportion of patients with fall-related injuries in the total survey population. We identified older patients in the injurious and non-injurious groups. Injury included moderate to severe classifications according to the categories and definitions provided in the systematic review conducted by Schewnk on behalf of the Prevention of Falls Network Europe [17, 18] as defined below, and no injury included no or minor injury classifications. Death was not included in the group because no adverse events directly led to death.

Fall-related injuries were classified as follows [18, 19]:

1. No injury: no physical injury detected.
2. Minor injury: minor bruises or abrasions not requiring health professional assistance and reduction in physical function (e.g., due to pain, fear of falling) for at least 3 days.
3. Moderate injury: wounds, bruises, sprains, and cuts requiring a medical/health professional examination, such as physical examination, radiography, and suture.
4. Severe injury: medically recorded fracture and head or internal injury requiring accident and emergency or inpatient treatment.

### Statistical methods

Statistical software (Statistical Package for the Social Sciences version 17.0) was used for data processing, with normally distributed continuous data expressed as the mean ± standard deviation. The enumeration data are expressed as frequency and percentage, and the chi-squared was performed for intergroup comparisons. ①For the chi-squared test of the fourfold table, if the theoretical number was T < 5 but T ≥ 1 and n ≥ 40, the continuity correction chi-squared was used for testing. ②We also implemented a chi-squared test for R × C tables by column merging.

All statistical tests were two-sided, and the significance level was set at *P* < 0.05. Based on the number of falls in the previous 3 months, patients were divided into recurrent fallers (at least one fall in the hospital or in the community) and single-fallers (no falls) [20] to analyze the statistical differences in the incidence of recurrent falls among the groups with different characteristics. We also analyzed the differences in the incidence of injurious falls among the different characteristic groups. We used odds ratio (OR) and 95% confidence interval (CI) to indicate the strength of the association between fall-related injuries and exposure in hospitalized older patients.

## Results

### Characteristics of older inpatients with falls

A total of 153 falls occurred in the older inpatients. Approximately 18.30% of the patients experienced an injurious fall, whereas 86.93% were single fallers during their hospitalization from 2018 to 2020. The age of the fallers ranged from 60 to 97 years (72.37 ± 8.13). A total of 59.48% of the fallers were men. A large proportion of the fallers maintained normal eyesight (93.46%), excreted normally (88.89%), and were awake (96.73%). Among the patients who fell, 20.92% were independent, and 79.08% were mildly, moderately, or severely dependent. Most patients who fell required assistance with mobility.

More than half of the fallers were either at the edge of the bed (38.56%) or toilet (28.76%). Most of the fallers (88.89%) were considered to have moderate or high fall risk based on Morse Fall Scale. The majority (66.01%) were with bed rails, but without auxiliary tools (92.81%) or catheters (83.66%). Almost a third (33.33%) of the fallers had comorbidities with tumors, coronary heart disease (16.99%), cerebrovascular disease (15.69%), and diabetes (10.46%). Responsible nurses were supervisors (41.83%) and with N3 level (26.20%). The general characteristics of falls among the older inpatients are shown in Table 3.

**Table 3:** The general characteristics of falls in older inpatients (*n* = 153)

Characteristics		Cases	Percentage (%)
Ward	Medical ward	68	44.44
	Surgical ward	38	24.84
	Geriatric ward	32	20.92
	gynecological ward	9	5.88
	The emergency ward	6	3.92
Occurrence year	2018	48	31.4
	2019	66	43.1
	2020	39	25.5
Age(year)	60–69	64	41.83
	70–79	53	34.64
	≥ 80	35	22.88
Gender	male	91	59.48
	female	62	40.52
Nomal eyesight	Yes	143	93.46
	No	10	6.54
Consciousness	awake	148	96.73
	confusion	5	3.27
Comorbidities	Tumor	51	33.33
	Coronary heart disease	26	16.99
	Cerebrovascular disease	24	15.69
	Diabetes	16	10.46
	Hypertension	14	9.15
	Waist, bone and joint injury	14	9.15
	Damage of liver and kidney function	11	7.19
	Diseases of he immune system	5	3.27
Serum electrolyte disorder	Yes	3	2.00
	No	150	98.00
Pain	Yes	3	2.00
	No	150	98.00
Fall-related injuries	no injury	76	49.67
	Minor injury	49	32.03
	Moderate injury	16	10.46
	Serious injury	12	7.84
Time interval	0am-4am	35	22.88
	4am-8am	34	22.22
	8am-12am	23	15.03
	12am-16 pm	24	15.69
	16 pm-20 pm	22	14.38
	20 pm-24 pm	15	9.80
Place	The edge of the bed	59	38.56
	Toilet	44	28.76
	The ward	26	16.99
	Inwards corridor	12	7.84
	Other areas outside the ward	11	7.19
Patient condition	Before or after toilet	74	48.4
	Bedside shift	41	36.9
	Walking	20	13.1
Normal excretion	Yes	136	88.89
	No	17	11.11
Medication	Antihypertensive drugs	49	32.03
	Hypoglycemic drugs	31	20.26
	Diuretics drugs	31	20.26
	Sedative drugs	18	11.76
	Antiarrhythmic drugs	5	3.27
With catheter	Yes	25	16.34
	No	128	83.66
ADL	No dependence	32	20.92
	Mild dependence	76	49.67
	Moderate dependence	25	16.34
	Severe dependence	20	13.07
Falling risk	No risk	3	1.96
	Low risk	14	9.15
	Moderate risk	78	50.98
	High risk	58	37.91
Falled in 3 months	Yes	20	13.07
	No	133	86.93
With bed rails	Yes	101	66.01
	No	52	33.99
With auxiliary tools (wheelchairs, boots, cane)	Yes	11	7.91
	No	142	92.81
Promary nurses grading	N0	1	0.70
	N1	20	13.10
	N2	92	60.1
	N3	40	26.20
Diploma	College	2	1.31
	Undergraduate	145	94.77
	master	6	3.92
Working years	Y < 5	14	9.15
	5 ≥ Y < 10	78	50.98
	Y ≥ 10	61	39.87
Positional title	Nurse	14	9.15
	Senior nurse	75	49.02
	Supervisor nurse	64	41.83

### Comparison between recurrent and single fallers

Compared to single fallers, older recurrent fallers were more likely to have cerebrovascular disease and ingest hypoglycemic drugs (*P* < 0.05). They were also at a higher risk for falling, and ≥ 3 fall times in 3 months; and their responsible nurses were more likely to be supervisors or with N3 level (*P* < 0.05). The analysis of the factors related to recurrent falls in older inpatients is presented in Table 4.

**Table 4:** Analysis of related factors of recurrent falling in older inpatients

Variables		Recurrent fallers (*n* = 20)	Single fallers(*n* = 133)	χ2	*P* value
With cerebrovascular disease	Yes	8	16	8.278	0.004
	No	12	115		
Hypoglycemic drugs	Yes	8	23	4.232	0.040
	No	12	110		
Falling risk	No or low or moderate risk	1	94	31.860	< 0.001
	High risk	19	39		
Fall times in 3 months	< 3	12	133	48.353	< 0.001
	≥ 3	8	0		
Primary nurses positional title	Nurse or senior nurse	7	82	5.076	0.024
	Supervisor nurse	13	51		
Primary nurses grading	N0 or N1 or N2	10	103	6.781	0.009
	N3	10	30		

### Odds ratio of fall-related injuries in older inpatients

When comparing non-injurious to injurious falls, significant statistical differences were found between fall characteristics and fall-related injuries. Factors that increased the risk of experiencing a fall-related injury were hypoglycemic drugs (OR 2.751; 95% CI 1.114–6.795), and nursing adverse events (OR 47.571; 95% CI 14.392–157.247). Older inpatients with bed rails (OR 0.437; 95% CI 0.190–1.005) or falling at the edge of the bed (OR 0.365; 95% CI 0.138–0.964) were less likely to be injurious than those without bed rails or not falling at the edge of the bed.

Fall risk was significantly correlated with severe fall-related injuries. Older inpatients with moderate (OR 5.517; CI 0.687–44.306) or high risk (OR 2.196; CI 0.251–19.219) risk were more likely to experience fall-related injuries than those with low risk. The ORs for fall-related injuries in older inpatients are presented in Table 5.

**Table 5:** OR of fall-related injuries in older inpatients

Variables		No or minor injury (*n* = 125)	Moderate or serious injury (*n* = 28)	χ2	*P* value	OR	95%CI
Nursing adverse events level	Vulnerabilities or no adverse events	111	4	68.039	< 0.001	47.571	14.392–157.247
	Adverse events	14	24				
With hypoglycemic drugs	Yes	21	10	5.065	0.024	2.751	1.114–6.795
	No	104	18				
With bed rails	Yes	87	14	3.917	0.048	0.437	0.190–1.005
	No	38	14				
Fall at the edge of the bed	Yes	53	6	4.369	0.037	0.365	0.138–0.964
	No	72	22				
Falling risk	No or low risk	16	1	6.497	0.039		
	Moderate risk	58	20			5.517	0.687–44.306
	High risk	51	7			2.196	0.251–19.219

## Discussion

To the best of our knowledge, few studies have focused on the incidence and clinical characteristics of fall-related injuries among older Chinese inpatients in a hospital setting. This cross-sectional study provides new evidence to rationalize the required attention of older inpatients’ fall-related injury prevention at a tertiary grade A hospital in Jinan, Shandong. This study could be considered some representativeness in mainland China by reporting the incidence of fall-related injuries, characteristics of falls, related factors of recurrent falls, and factors that increase the risk of fall-related injury.

### Incidence of fall-related injuries

Our results showed that the incidence of fall-related injuries was 18.30%, which was lower than previous findings. Different definitions used in different hospitals reported a prevalence of injurious falls in older adult patients of 23.80 ~ 89.74% in mainland China [6–9, 21]. For example, Feng et al. [6] reported that the fall-related injury incidence among a retrospective analysis of 495 inpatients aged ≥ 60 years was 42.42% in Zhejiang Province AERS from 2009 to 2012. Recently, an investigation by Xu et al. [8] of 238 inpatients aged ≥ 60 years reported that the prevalence of fall-related injuries was 63.21% in Jiangsu Province Hospital from 2017 to 2020.

Fall-related injuries have been studied in other countries, with different results. Moreover, approximately a tenth of adults aged ≥ 65 years were injured by falls in 2018 according to statistics from the Behavioral Risk Factor Surveillance System in the United States [22]. Approximately 30% of falls in hospitals result in injuries, with 1%–5% leading to severe injuries in 206,350 falls from a total of 472 hospitals in National Reporting and Learning System (NRLS) for patient safety incidents in England and Wales [23].

Generally, the incidence of fall-related injuries in this study was minor among studies conducted in hospitals worldwide. These results can be explained as follows:

First, we used the total number of inpatients aged ≥ 60 years who fell in a hospital as the denominator to calculate the incidence of fall-related injuries. Since the fallers only included older patients from multiple geriatric departments in the same hospital, we expected a lower incidence of injurious falls than in other studies that included different hospitals. Second, the strategy of encouraging non-punitive active reporting makes the denominator fully reported. Third, the comparison results may have been affected by the different definitions of fall-related injuries and different areas. For example, annual national reporting on fall incidents in hospitals in the UK NRLS requires the measurement of falls resulting in patient harm. The numerator is the number of in-hospital falls resulting in low, moderate, and severe harm and death [23], whereas our study included moderate-to-severe injury for injurious falls. Fourth, patient characteristics, such as age and health status, limited the ability to directly compare findings. Fifth, in early 2020, coronavirus disease 2019 ravaged many countries worldwide, including China, resulting in fewer hospitalizations, falls, and fall-related injuries. Sixth, based on an overview of continuous care interventions to prevent falls in the guidelines [24–27], we developed fall prevention strategies, including risk factor screening, assessment, and management, to ensure timely detection and comprehensive care of falls by addressing existing health problems, improving risk status, or delaying progress.

### Rates for recurrent falls and factors associated with recurrent falls

Compared with single fall incidents, recurring falls usually lead to more serious consequences, such as immobility, long-term hospitalization, and even death [28]. In this study, the crude rate for recurrent falls was 13.07%, which was comparatively higher than the recurrent fall rates ranging from 4.6% to 10.4% reported in previous studies [29, 30]. This difference may be explained by the older participants in our sample (age ≥ 60 years) compared to previous studies that comprised younger adults (age ≥ 40 years).

As shown in Table 3, recurrent falls were associated with cerebrovascular disease, use of hypoglycemic drugs, high risk of falls, number of falls ≥ 3 within 3 months, and the responsible nurse being a supervisor or at N3 level. Many studies have reported similar results [31–34]. In these studies, diseases that interfere with mobility and fall risk were associated with recurrent falls. However, Li et al. [21] reported that a high risk of falls was not significantly associated with recurrent falls, which is contrary to our result. Possible explanations for this might be the use of different fall risk assessment tools and sample cases. The incidence density was highest within the 3 months after the first injurious fall [35]. Therefore, the different study periods in each case might be the reason for these conflicts. Furthermore, coordination and balance issues place older people at an increased risk of recurrent falls and fall-related injuries [36]. Muscle and gait were also reported as predictors of falling based on the American Geriatrics Society/British Geriatrics Society Clinical Practice Guideline for Prevention of Falls in Older Persons (2010) [26]. However, these factors were not assessed in this study. Clinical practice guidelines recommend [37] that older people should be screened for risk of falls at least once a year; balance disorders, gait [25], and movement limitations are necessary conditions for screening for risk of falls.

Unexpectedly, older patients cared for by senior primary nurses were more at risk to recurrent falls than those cared for by junior primary nurses. This outcome is contrary to that of Zhang, who found that senior nurses paid more attention to the risk of inpatient falls and effectively avoided the occurrence [38]. This inconsistency may be because there were relatively more senior nurses in the whole hospital; therefore, the differences were more significant.

### Factors associated with fall-related injuries

In the present study, falls reported as adverse events were more at risk to fall-related injuries. This result was further confirmed by reports of nursing adverse events in hospitals adhering to the principle of non-punitive and active reporting. Fortunately, it is feasible to prevent falls as adverse events and fall-related adverse outcomes [39, 40].

In the current study, older inpatients with bed rails or falling at the edge of the bed were less likely to experience fall-related injuries than those without bed rails or not falling at the edge of the bed. However, the role of bed railings in preventing fall-related injuries remains controversial. Hignett and Masud [41]reported that there was no clear evidence that elevated bed railings could prevent fall-related injuries. Instead, they suggested that the bed should be reduced to the ground height when the patient is lying in bed. Rensburg et al. [42] also showed that inappropriate use of bed rails contributed to moderate and major fall-related injuries. Their results differed from those presented here, which suggests that more studies need to be conducted to confirm the role of bed rails in preventing fall-related injuries.

Compared with the existence of no or low risk, the current study found that the risk of more severe fall-related injuries increased 5.517 times when a moderate risk existed and 2.196 times if a high risk existed. Following the present results, previous studies have demonstrated that high fall risk independently predicted fall-related injury [43]. It is difficult to explain that a moderate risk of falls is likely to be associated with a greater risk of fall-related injury, but it might be related to the risk of patient falls being underestimated if fall risk assessment tools are used inappropriately, and patients at a high risk of falling may consciously take some fall prevention measures. In addition to comprehensive risk reduction strategies tailored for specific high-risk groups [44], individualized multifactorial risk assessment for fall-related injury prevention should not be ignored [25, 45, 46], rather than an overall assessment score of fall risk factors using risk prediction tools [47]. A scientific and comprehensive assessment that can be used as a standard will be a direction for future studies.

In our study, fallers using hypoglycemic drugs were more likely to experience fall-related injuries than fallers not using hypoglycemic drugs. This concurred with the observations by Yau et al. [33], who showed that patients with diabetes receiving insulin therapy were almost three times more likely to experience fall-related injuries than patients with diabetes without insulin treatment. Therefore, for older patients with diabetes, maintaining appropriate blood glucose control and improving balance can reduce the risk of fall-related injuries. In this study, only five common drugs that could lead to falls in hospitalized older patients were studied. In the future, studies of polypharmacy and other medications that increase the risk of falls can provide additional evidence for the prevention of falls in older adults, since polypharmacy and potentially inappropriate medication use for older patients [48] may increase the risk of drug-related injurious falls [49].

Analyzing the factors related to fall-related injuries is helpful in investigating the reasons that are associated with such injuries. It is crucial to consider methods that can prevent fall-related injuries based on these factors. In fact, Blain et al. found that appropriate multidisciplinary fall risk and comprehensive geriatric assessment, including physical evaluation and social support, could significantly reduce serious and moderate fall-related injuries [50]. Based on randomized clinical trials published in 2019 [51], long-term exercise (≥ 1 year) reduced injurious falls, especially lowering the fracture risk in people aged ≥ 60 years.

Further studies on fall-related injury prevention in hospitals are required to reduce the incidence of falls. One study found [52] that older patients had contradictory views on fall injury prevention. The determinants are, on the one hand, the perceived need for safety and freedom from harm and, on the other hand, the need to maintain autonomy and independence. The willingness of older patients to comply with fall prevention depends on these needs. Encouraging patients and their families to participate in patient safety is one of the top 10 patient safety goals [53]. Strategies on how to encourage patients and their families to participate in injurious fall prevention and cooperate with the hospital for comprehensive assessment is a topic for future studies.

### Limitations

This study has some limitations. First, the sample size was limited because only one hospital was involved in this study, and variabilities in the diverse locations of falls, methods of collecting information, and definitions of fall-related injuries in the study design limit the ability to directly compare findings. It is therefore difficult to generalize the findings. Second, we could not confirm the risk factors for falling because of the lack of a control group. A cross-sectional survey cannot determine the causal relationship between the results and explanatory variables. Third, there may be recall bias in the way the data were collected, leading to under-reporting of falls. Fourth, non-punitive and active reporting may result in falls that do not cause injury being less likely to be reported than falls that do. Fifth, the clinical characteristics mentioned in the study were not comprehensive, and other modifiable factors associated with fall-related injuries (e.g., muscle, gait, balance disorders, and polypharmacy) were not assessed.

## Conclusions

Older inpatient falls are an ongoing challenge in hospitals in China. This study illustrated the use of a fall AERS in conjunction with data on fall-related injuries to investigate the incidence and clinical characteristics of fall-related injuries and recurrent falls among inpatients aged ≥ 60 years in a tertiary grade A hospital in Shandong Province. We found that the incidence of fall-related injuries among inpatients aged ≥ 60 years remained at a minor level. However, complex patient characteristics and circumstances can contribute to fall-related injuries.

This study provides new evidence on fall-related injuries in older inpatients in China. Based on the factors found in this study, regular fall-related injury epidemiological surveys that investigate the reasons associated with the injuries were crucial when considering intervention measures that could refine fall-related injuries. More prospective studies should be conducted with improved and updated multidisciplinary fall risk assessment and comprehensive geriatric assessment as part of a fall-related injury prevention protocol.

## Data Availability

The datasets generated and/or analyzed during the current study are not publicly available due to subject confidentiality but are available from the corresponding author on reasonable request.

## Abbreviations

AERS: Adverse event reporting system
ADL: Activity of daily living
OR: Odds ratio
CI: Confidence interval
NRLS: National Reporting and Learning System
